# Similar bacterial communities on healthy and injured skin of black tip reef sharks

**DOI:** 10.1186/s42523-019-0011-5

**Published:** 2019-09-17

**Authors:** Claudia Pogoreutz, Mauvis A. Gore, Gabriela Perna, Catriona Millar, Robert Nestler, Rupert F. Ormond, Christopher R. Clarke, Christian R. Voolstra

**Affiliations:** 1 Red Sea Research Center, Biological and Environmental Science and Engineering Division (BESE), King Abdullah University of Science and Technology (KAUST), Thuwal, 23955 Saudi Arabia; 2 Marine Conservation International, South Queensferry, Edinburgh, Scotland, UK; 3 Centre for Marine Biodiversity & Biotechnology, Heriot-Watt University, Riccarton, Edinburgh, Scotland, UK; 4 Veterinär-Physiologisch-Chemisches Institut, University of Leipzig, 04107 Leipzig, Germany; 5 Faculty of Marine Sciences, King Abdulaziz University, Jeddah, 21589 Saudi Arabia; 6 Marine Research Facility, North Obhur, Jeddah, Saudi Arabia; 7 Department of Biology, University of Konstanz, 78457 Konstanz, Germany

**Keywords:** Skin microbiota, *Pseudoalteromonas*, *Psychrobacter*, Lesion, Injury, Wound healing, Immunity, Elasmobranch, *Carcharhinus*

## Abstract

**Background:**

Sharks are in severe global decline due to human exploitation. The additional concern of emerging diseases for this ancient group of fish, however, remains poorly understood. While wild-caught and captive sharks may be susceptible to bacterial and transmissible diseases, recent reports suggest that shark skin may harbor properties that prevent infection, such as a specialized ultrastructure or innate immune properties, possibly related to associated microbial assemblages. To assess whether bacterial community composition differs between visibly healthy and insulted (injured) shark skin, we compared bacterial assemblages of skin covering the gills and the back from 44 wild-caught black-tip reef sharks (*Carcharhinus melanopterus*) from the Amirante Islands (Seychelles) via 16S rRNA gene amplicon sequencing.

**Results:**

Shark skin-associated bacterial communities were diverse (5971 bacterial taxa from 375 families) and dominated by three families of the phylum *Proteobacteria* typical of marine organisms and environments (*Rhodobacteraceae*, *Alteromonadaceae*, *Halomonadaceae*). Significant differences in bacterial community composition of skin were observed for sharks collected from different sites, but not between healthy or injured skin samples or skin type (gills vs. back). The core microbiome (defined as bacterial taxa present in ≥50% of all samples) consisted of 12 bacterial taxa, which are commonly observed in marine organisms, some of which may be associated with animal host health.

**Conclusion:**

The conserved bacterial community composition of healthy and injured shark skin samples suggests absence of severe bacterial infections or substantial pathogen propagation upon skin insult. While a mild bacterial infection may have gone undetected, the overall conserved bacterial community implies that bacterial function(s) may be maintained in injured skin. At present, the contribution of bacteria, besides intrinsic animal host factors, to counter skin infection and support rapid wound healing in sharks are unknown. This represents clear knowledge gaps that should be addressed in future work, e.g. by screening for antimicrobial properties of skin-associated bacterial isolates.

**Electronic supplementary material:**

The online version of this article (10.1186/s42523-019-0011-5) contains supplementary material, which is available to authorized users.

## Background

Sharks are in global decline due to intensive human exploitation. Most large-bodied species have been reduced to an estimated less than 10% of their original populations [1–5]. As a consequence, many of them are now considered threatened or endangered [6, 7]. As sharks occupy critical ecological roles in marine ecosystems [6, 8, 9], many Marine Protected Areas (MPAs) are now being managed with the need to protect sharks in mind, and some ten countries have designated their territorial waters ‘Shark Sanctuaries’, with all shark species afforded protection [10].

Anthropogenic pressures from targeted fishing and bycatch constitute the main threats to global shark populations [1–5]. However, other potential threats include susceptibility of sharks to bacterial infection and/or transmissible diseases, which seem to increase in marine organisms over recent decades [11]. While only a few documented infections of sharks in the wild are available [12, 13], sharks can often be observed bearing open wounds without any obvious sign of infection [14, 15]. In contrast, increased frequency and severity of bacterial and/or eukaryotic infection has been described for sharks in captivity, in particular when kept at high densities [16–23].

Like all other animals, sharks should be considered metaorganisms, i.e. animals hosts associated with a diverse microbial community collectively termed the microbiome [24, 25]. This microbiome typically consists of prokaryotes (Bacteria, Archaea), eukaryotes (fungi, protists, algae), and viruses [26, 27]. Skin in particular constitutes a large habitat for animal-associated bacteria, creating an abundance of niches for unique microbial communities [28]. Environmental stress can lead to a disturbance of associated microbiota, the structural and functional disruption of the entire community and, ultimately, disease [29, 30]. Consequently, skin diseases [31–37] as well as mechanic insult, disruption, or irritation of skin [38] may cause distinct changes in the associated bacterial microbiome.

**Fig. 1:**
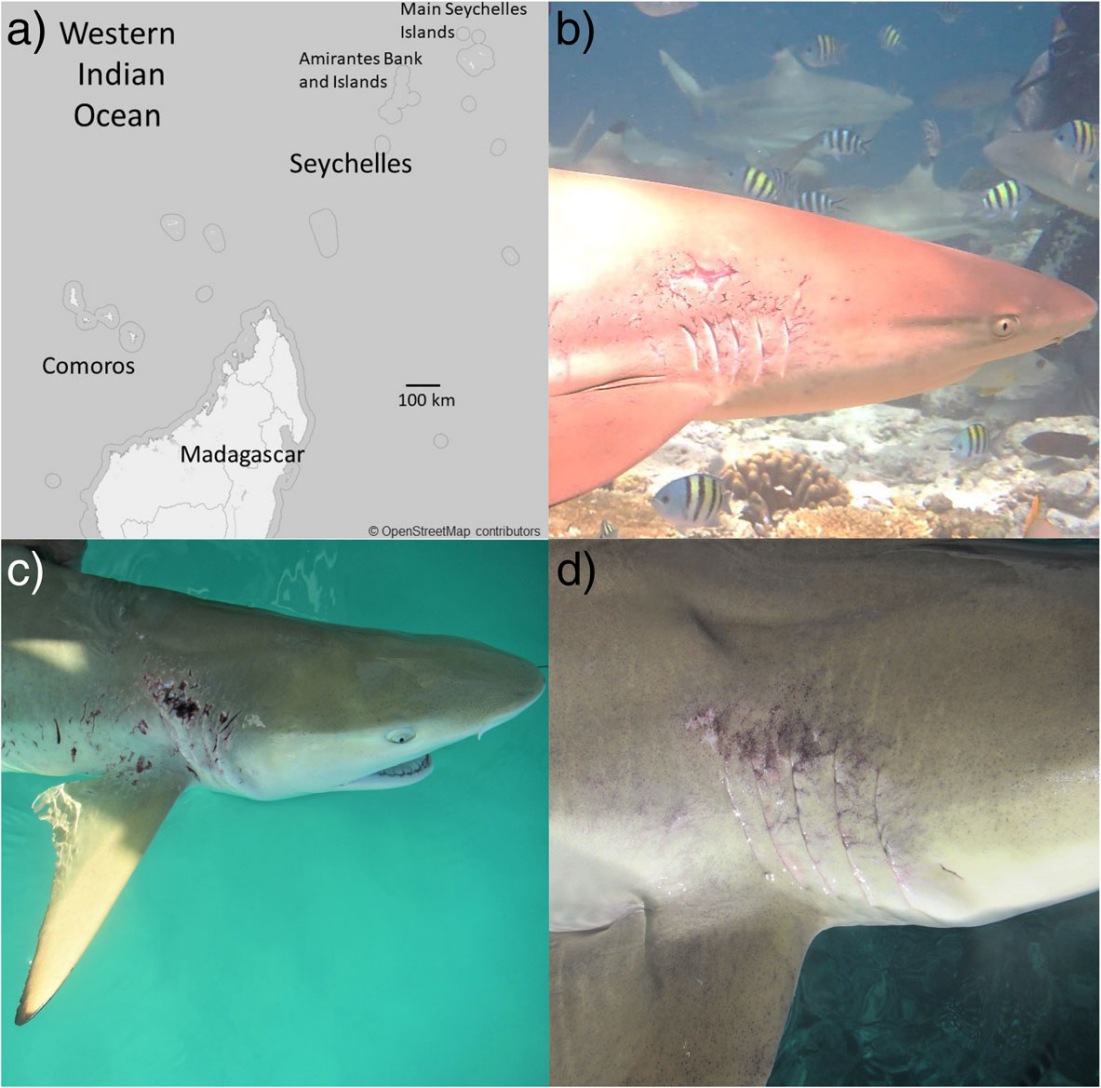
Black-tip reef shark (*Carcharhinus melanopterus*) sampling and skin insults. **a** Shark skin swab samples were collected at five sites in the Amirante Islands, Seychelles. **b** Black-tip reef sharks can often be observed exhibiting deep open skin injuries. **c-d** Representative photographs of insults on the skin covering the gills in black-tip reef sharks. Photographs taken (**b**) of a free-swimming shark at one of the sample sites, (**c**) and (**d**) during shark capture and sample collection

The black-tip reef shark (*Carcharhinus melanopterus*), a medium-sized and relatively common Indo-Pacific predator [39], can often be observed in the wild bearing severe skin insults, such as deep open wounds (Fig. 1b; [15]). At the same time, this species seemingly exhibits a highly developed capacity for rapid wound healing after skin injury [15]. In this context, it is important to understand the contribution of the resident bacterial community on the skin to such properties. In the present study, we therefore investigated bacterial community structure of skin samples from a population of black-tip reef sharks (*Carcharhinus melanopterus*) in the Amirante Islands (Seychelles). Of the sharks sampled, a proportion were noticeably affected by skin insults (lesions) and surface irregularities, especially around and behind the gills. This allowed us to profile bacterial communities associated with visibly healthy skin and compare them to the skin from conspecifics bearing such injuries (Fig. 1c-d), so as to determine whether bacterial community shifts align with healthy and insulted skin samples of black-tip reef sharks.

## Results

### Bacterial community composition of black-tip reef shark skin

To assess bacterial community composition of healthy and compromised skin areas of the gills and the back, we conducted amplicon sequencing of the V5 and V6 region of the bacterial 16S rRNA gene from wild-caught sharks from the Amirante Islands in the Seychelles (Fig. 1a). In total, 88 skin samples from 44 black-tip reef sharks (one mucus swab sample each from the skin covering over and around the gills and one from the back of each individual shark) were collected from five sites in the study area. Overall, 28 of the sampled sharks were visibly healthy and 16 exhibited marked insult(s) on the skin around the gill area (Table 1, Fig. 1c-d). A total of 18,022,131 16S rRNA gene amplicon sequences were determined, distributed over the 88 samples. After quality checks and removal of unwanted sequences, 2,034,047 sequences with an average length of 293 bp remained, and were clustered at 97% similarity into 5971 distinct bacterial Operational Taxonomic Units (OTUs; ‘taxa’) from 375 bacterial families (Additional file 4: Table S1, Additional file 5: Table S2). Plateauing rarefaction curves suggest sufficient sampling effort, higher variation in numbers of OTUs in gill samples than in back samples, and higher numbers of OTUs per sample for the sampling site North Side; for details, see Additional file 2: Figure S1).

**Table 1:** Overview of shark samples collected

	Healthy	Skin-insulted	No. of gill and back samples
Number of sharks	27	17	Each 44
Number of sharks per site			
West Ressource (St. Joseph)	5	7	Each 12
East Ressource (St. Joseph)	8	3	Each 11
Fouquet (St. Joseph)	5	2	Each 7
Benjamin (St. Joseph)	0	3	Each 3
North Side (D’Arros)	9	2	Each 11

The majority of bacterial sequences on the phylum level were assigned to *Proteobacteria* (63.4%), *Bacteroidetes* (24.0%), *Actinobacteria* (6.1%), *Firmicutes* (5.3%), and others (1.2%). On the class level, most sequences were assigned to *Gammaproteobacteria* (34.8% of total sequences and 54.9% of *Proteobacteria*), *Alphaproteobacteria* (24.6% of total sequences and 38.8% of *Proteobacteria*), *Acidimicrobia* (3.6% of the total), and *Bacilli* (3.3% of the total); remaining bacterial sequences were assigned to low abundance classes, cumulatively making up 33.7% of the total. Overall, the three most abundant bacterial families observed (ranked by realitve abundance) included the *Rhodobacteraceae* (*Alphaproteobacteria*: *Rhodobacterales*; on average contributing 16.0 and 13.2% of the total bacterial community on the skin around the gills and back skin, respectively), *Alteromonadaceae* (*Gammaproteobacteria*: *Alteromonadales*; 10.7 and 12.1% of the total around gills and backs, respectively), and *Halomonadaceae* (*Gammaproteobacteria*: *Oceanospirillales*; 4.8 and 5.4% of the total around gills and backs, respectively). Other bacterial families individually contributed around 5% or less to the total (Additional file 4: Table S1).

**Fig. 2:**
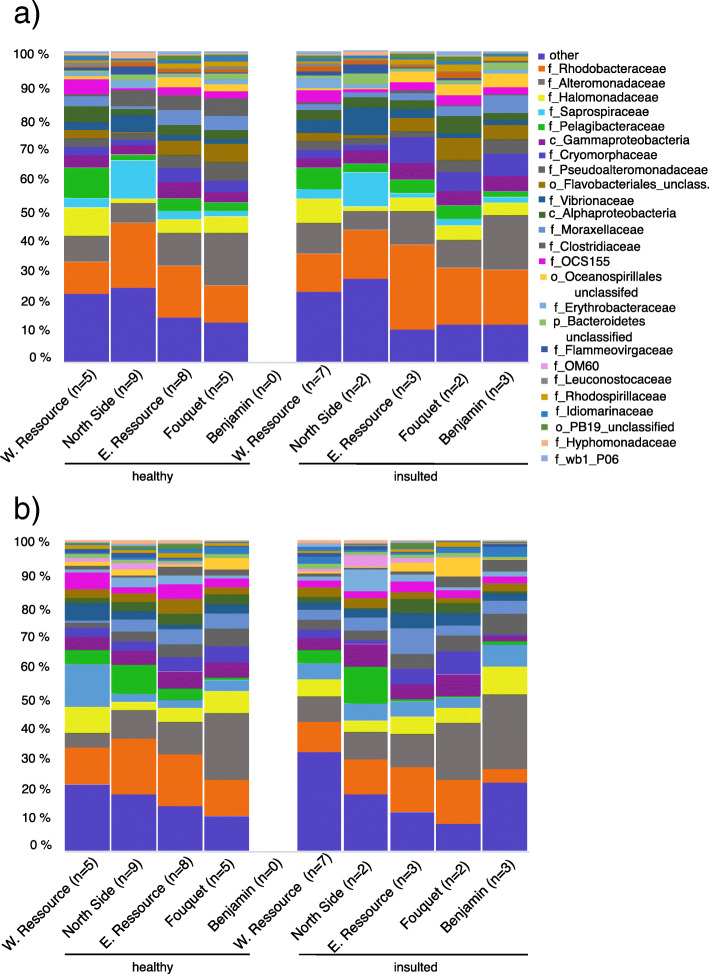
Family-level stacked bar plots showing bacterial community composition of healthy and insulted skin samples of black-tip reef sharks (*Carcharhinus melanopterus*) collected at different sites in the Amirante Islands, Seychelles. **a** Samples from the skin around the gill area. **b** Samples from the skin on the back of the shark. There are no statistically significant differences at OTU level for health state (‘healthy’, ‘insulted’; PERMANOVA, Pseudo-F = 1.1031; *p* = 0.2646), and location on skin (‘gill’, ‘back’; PERMANOVA, Pseudo-F = 1.316, *p* = 0.2839). Community composition was significantly different at OTU level between study sites (PERMANOVA, Pseudo-F = 4.1429, *p* < 0.0001)

Overall, the bacterial community composition was uneven (Simpson’s Evenness of the bacterial communities mean ± SE = 0.07 ± 0.003) (Table 2). No difference was observed in the most abundant bacterial OTUs between skin samples from visibly healthy and lesioned gill areas or control samples from the back (PERMANOVA; F = 83,592, R^2^ = 0.0963, *p* = 0.5657, Table 3 a; Fig. 2; for bar plots showing bacterial community composition of individual samples, see Additional file 3: Figure S2). The core microbiome at a cut-off of 80% (i.e., present in 80% of samples) consisted of the two most abundant OTUs, i.e. OTU00001 (*Rhodobacteraceae* sp.) and OTU00002 (*Alteromonas* sp.). At a less stringent cut-off of 50% (i.e., present in 50% of samples), the core microbiome consisted of 11 OTUs, more specifically OTUs 00001–00006 (*Rhodobacteraceae* sp., *Alteromonas* sp., *Pelagibacteraceae* sp., *Flavobacteriales* sp., *Vibrionales* sp., OCS155 sp.), OTUs 00010–00011 (*Oceanospirillales* sp., *Psychrobacter pacificensis*), 14 (*Flavobacteriaceae* sp.), OTUs 16 (*Pseudoalteromonoas porphyrae*) and 19 (*Halomonadaceae* sp.) (OTUs 00001–00006, 00010, 00011, 00014, 00016, and 00019).

**Table 2:** Statistics of 16S rRNA gene amplicon sequencing, and richness and diversity indices of bacterial communities associated with visibly healthy and infected skin around the gills and visibly healthy skin on the back of black-tip reef sharks (*Carcharhinus melanopterus*) collected in the Amirante Islands (Seychelles). BD = samples from skin on the back; GD = samples from skin around the gills; F = female; M = male; H = visibly healthy sharks; D = sharks with infected skin around the gills

Sample	Chao1 Index	Inverse Simpson Index	Simpson’s Evenness	Number of Seqs
a) Skin around the gills				
CM01_GD_F_H	829.11	68.73	0.1	22,837
CM02_GD_F_H	1225.4	113.21	0.1	22,612
CM03_GD_F_I	856.24	38.5	0.05	23,567
CM04_GD_F_H	585.64	43.1	0.09	23,337
CM05_GD_F_I	670.49	102.7	0.18	21,951
CM06_GD_F_I	579.88	53.57	0.11	23,006
CM07_GD_F_I	1428.6	106.33	0.09	23,823
CM08_GD_M_H	1037.1	98.46	0.11	23,578
CM09_GD_F_H	1501.9	92.43	0.07	23,743
CM10_GD_F_H	3181.1	123.1	0.05	24,111
CM11_GD_F_H	1088.9	8.05	0.01	23,495
CM12_GD_F_I	1612.1	133.24	0.1	23,782
CM13_GD_F_H	1853.1	130.33	0.09	23,751
CM14_GD_F_H	1476.1	14.87	0.01	23,674
CM15_GD_F_H	1837.9	109.52	0.07	23,954
CM16_GD_M_H	3442.4	100.96	0.04	24,120
CM17_GD_F_H	2021.6	183.98	0.1	23,814
CM18_GD_M_H	546.05	51.66	0.11	22,227
CM19_GD_M_H	790.74	53.95	0.08	23,413
CM20_GD_M_I	508.86	30.46	0.07	22,719
CM21_GD_F_I	854.37	25.41	0.04	24,060
CM22_GD_F_H	623.35	28.13	0.05	23,716
CM23_GD_M_H	567	34.65	0.07	22,839
CM24_GD_F_H	554.29	33.12	0.07	21,255
CM25_GD_M_H	486.78	39.75	0.09	23,369
CM26_GD_M_H	615	31.26	0.07	23,417
CM27_GD_M_I	792	34.57	0.05	23,625
CM31_GD_F_I	509.08	26.13	0.06	23,473
CM32_GD_M_H	437	14.2	0.04	22,970
CM33_GD_F_H	567.85	34.52	0.07	23,452
CM34_GD_F_I	904.58	31.56	0.04	23,506
CM35_GD_M_H	559.91	29.73	0.07	22,633
CM36_GD_F_H	574.42	18.28	0.04	21,942
CM37_GD_M_H	755.85	38.93	0.06	21,908
CM38_GD_F_H	671.21	13.45	0.02	23,638
CM39_GD_M_I	641.87	27.18	0.05	23,472
CM40_GD_M_I	620.64	26.61	0.05	23,623
CM41_GD_M_I	609.35	24.07	0.05	23,727
CM42_GD_F_I	236	29.44	0.15	21,194
CM43_GD_F_I	786.4	39.58	0.06	23,589
CM44_GDL_F_I	1090.6	63.38	0.08	23,187
CM45_GD_F_H	921.89	28.71	0.04	22,768
CM47_GD_F_I	1266.1	66.5	0.06	22,222
CM48_GD_F_I	791.26	39.77	0.06	23,720
b) Skin from the back				
CM01_BD_F_H	424.05	22.19	0.07	22,718
CM02_BD_F_H	544.43	29.67	0.07	23,087
CM03_BD_F_I	399.3	61.82	0.18	22,542
CM04_BD_F_H	459.24	30.95	0.07	23,172
CM05_BD_F_I	502.25	51.18	0.11	21,367
CM06_BD_F_I	691.78	74.37	0.12	20,901
CM07_BD_F_I	1615.9	143.2	0.1	23,496
CM08_BD_M_H	1054.4	126	0.13	23,287
CM09_BD_F_H	501.1	51.88	0.12	21,079
CM10_BD_F_H	1013	94.43	0.11	23,249
CM11_BD_F_H	963.75	84.59	0.11	23,299
CM12_BD_F_I	908.87	137.11	0.18	23,033
CM13_BD_F_H	2338.8	150.65	0.08	23,996
CM14_BD_F_H	2374.2	226.81	0.12	23,992
CM15_BD_F_H	2541.1	103.63	0.06	24,112
CM16_BD_M_H	3500.8	81.1	0.03	24,155
CM17_BD_F_H	2987.9	144.14	0.07	23,952
CM18_BD_M_H	454.36	38.21	0.09	22,717
CM19_BD_M_H	564.5	37.43	0.08	23,157
CM20_BD_M_I	507.23	48.92	0.11	21,976
CM21_BD_F_I	457.16	31.31	0.08	23,450
CM22_BD_F_H	480.28	34.59	0.08	22,383
CM23_BD_M_H	549.26	28.39	0.06	23,067
CM24_BD_F_H	650.16	33.82	0.06	22,486
CM25_BD_M_H	449.22	36.44	0.09	22,757
CM26_BD_M_H	533.81	35.57	0.08	23,177
CM27_BD_M_I	591.23	29.23	0.06	23,476
CM31_BD_F_I	531.63	29.43	0.06	22,829
CM32_BD_M_H	442.83	24.99	0.06	22,781
CM33_BD_F_H	519.56	29.86	0.06	23,381
CM34_BD_F_I	522	29.84	0.06	22,841
CM35_BD_M_H	598.38	30.95	0.06	22,562
CM36_BD_F_H	578.88	29.27	0.06	23,251
CM37_BD_M_H	408.6	13.95	0.04	24,023
CM38_BD_F_H	518	27.97	0.06	23,295
CM39_BD_M_I	506.66	26.52	0.06	23,406
CM40_BD_M_I	499.15	22.4	0.05	23,491
CM41_BD_M_I	614.56	23.54	0.04	23,770
CM42_BD_F_I	554.77	21.66	0.04	23,273
CM43_BD_F_I	843.32	30.37	0.04	23,538
CM44_BD_F_I	571.12	20.85	0.04	22,610
CM45_BD_F_H	549.06	18.33	0.04	22,963
CM47_BD_F_I	1024	26.25	0.03	22,639
CM48_BD_F_I	682.5	25.46	0.04	22,492

**Table 3:** Results of global and pairwise test statistics comparing differences in composition of bacterial communities associated with visibly healthy and insulted skin around the gills and visibly healthy skin on the back of black-tip reef sharks (*Carcharhinus melanopterus*) collected in the Amirante Islands (Seychelles). **a)** PERMANOVA results under unrestricted permutation to assess statistical differences of location (gills vs. back) of skin bacterial communities. **b)** Global PERMANOVA results with permutation of residuals under a reduced model to assess statistical differences of sampling site (‘site’), health status (‘health’), and sex of shark (‘sex’) on bacterial community composition on skin around the gills. **c)** Global PERMANOVA resutls with permutation of residuals under a reduced model to assess statistical differences of sampling site (‘site’), health status (‘health’), and sex of shark (‘sex’) on bacterial community composition on skin on the back. **d)** Summary of ANOSIM pairwise tests for ‘site’. Global *R* = 0.551, significance level *p* < 0.0001

PERMANOVA table of results							
a) Pairwise PERMANOVA (gills vs. back)							
Terms added sequentially (first to last)							
Df		Su	SS	MS	F.Model	R2	Pr(>F)
Skin	1	0.2019	0.20193	0.20193	0.8359	0.0963	0.5657
Residuals	86		20.7746	0.24157	0	99,037	
Total	87		20.9765		1	0	
b) global PERMANOVA for gill samples							
Terms added sequentially (first to last)							
		Df	SS	MS	F.Model	R2	Pr(>F)
Site		4	3.787	0.94675	5.5281	0.3651	0.0001
Health		1	0.2015	0.20145	1.1763	0.01942	0.2666
Sex		1	0.1692	0.16915	0.9877	0.01631	0.4169
Site:Health		3	0.4111	0.13705	0.8002	0.03964	0.7672
Site:Sex		4	0.7226	0.18064	1.0548	0.06966	0.3818
Health:Sex		1	0.1146	0.11461	0.6692	0.01105	0.7496
Residuals		29	4.9666	0.17126		0.47882	
Total		43	10.3725			1	
c) global PERMANOVA for back samples							
Terms added sequentially (first to last)							
		Df	SS	MS	F.Model	R2	Pr(>F)
Site		4	3.5699	0.89246	4.9904	0.34319	0.0001
Health		1	0.195	0.19504	1.0906	0.01875	0.3035
Sex		1	0.2623	0.26227	1.4665	0.02521	0.1232
Site:Health		3	0.4142	0.13808	0.7721	0.03982	0.8355
Site:Sex		4	0.6674	0.16684	0.9329	0.06416	0.5962
Health:Sex		1	0.1071	0.10709	0.5988	0.01029	0.853
Residuals		29	5.1862	0.17884		0.49858	
Total		43	10.4021				
d) ANOSIM for gill/back samples							
Pairwise Tests							
Groups		R Stats	Sig. Level			Act. Perm.	
StJos_WRes, D’Arros_North		0.8415/0.6811	0.001/0.001			9999	
StJos_WRes, StJos_ERes		0.3809/0.4359	0.002/0.001			9999	
StJos_WRes, StJos_Fouq		0.3692/0.2233	0.002/0.024			9999	
StJos_WRes, StJos_Ben		0.4525/0.1103	0.001/0.241			9999	
D’Arros_North, StJos_ERes		0.8476/0.8237	0.001/0.001			9999	
D’Arros_North, StJos_Fouq		0.8923/0.7936	0.001/0.001			9999	
D’Arros_North, StJos_Ben		0.9164/0.7095	0.003/0.007			9999	
StJos_North, StJos_Fouq		0.01162/0.01265	0.364/0.371			9999	
StJos_ERes, StJos_Ben		0.1076/0.3354	0.270/0.086			9999	
StJos_Fouq, StJos_Ben		0.0119/0.1746	0.436/0.184			9999

### Shark skin microbiomes differ between collection sites, but not between location on skin or condition

**Fig. 3:**
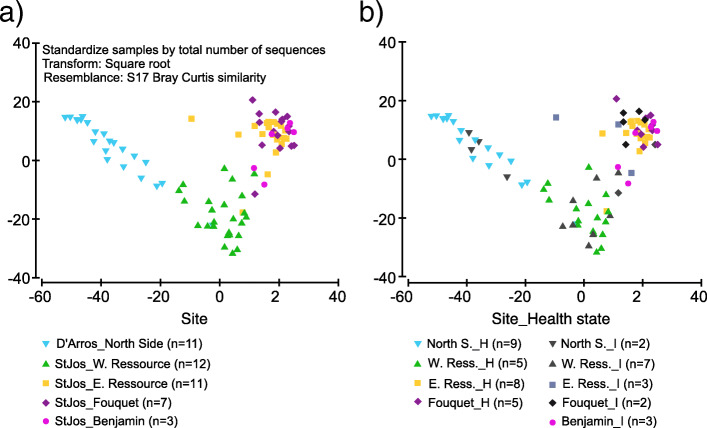
Principal Coordinate plots based on bacterial community composition of skin samples of the black-tip reef shark (*Carcharhinus melanopterus*; gill and back skin samples pooled). **a** Color-coded for collection sites; **b** Color-coded for collection site and shark health state. H = visibly healthy, I = insulted

To assess whether community composition of skin-associated bacterial communities differed between health states of shark skin (visibly healthy and insulted) of black-tip reef sharks, and across the five sites in the Amirante Islands, Seychelles, we conducted a Permutational Analysis of Variance (PERMANOVA) on microbiome assemblages using the *adonis* function in the R package vegan [81]. Significant differences for shark skin bacterial communities were apparent for collection site, both for samples from gills (*adonis* PERMANOVA, Pseudo-F = 5.5281, R^2^ = 0.3561, *p* < 0.0001, Table 3 b) and the back (*adonis* PERMANOVA; Pseudo-F = 4.9904, R^2^ = 0.34319, *p* < 0.0001, Table 3 c). There were however no significant differences between the two health states of skin samples taken from gills (PERMANOVA, Pseudo-F = 1.1763; R^2^ = 0.01942, *p* = 0.2666, Table 3 b), nor between those and samples from the back areas (PERMANOVA, Pseudo-F = 1.0906, *p* = 0..3035, Table 3 c). No significant interactions between any of the factors ‘health’, ‘site’, or ‘sex’ were observed for skin covering the gills (Table 3 b) or skin on the back (Table 3 c). However, pairwise ANOSIM comparisons for gill and back samples from different sites subsequently demonstrated significant differences in skin bacterial communities for the majority of sites (Table 3 d). No significant differences were observed between male and female sharks (Table 3 b, c). Principal Coordinate plots support the statistical analyses, showing the samples clustering by site, but not by health state (Fig. 3a,b).

In order to identify bacterial OTUs with differential abundance between study sites and in relation to skin location, a two-way ANOVA was conducted (Additional file 5: Table S2). It identified a total of 840 OTUs differentially abundant between collection sites, including 18 out of the 20 most abundant OTUs (Additional file 5: Table S2). Among these, several core microbiome taxa (OTU00001, OTU00002, OTU00004, OTU00010, OTU00011, OTU00014, OUT00016, OTU00019) exhibited higher relative abundances on sharks caught at sites located off of St. Joseph Atoll (i.e., East Ressource, Fouquet, and Benjamin), in contrast to the sites closer to the island d’Arros (i.e., North Side, West Ressource). Only one bacterial taxon (OTU00005; *Vibrionales* sp.) was more abundant on the skin of sharks collected at West Ressource and North Side compared to the other three sites off St. Joseph. One OTU (OTU00006; *OCS155* sp.) was more abundant at the four sites belonging to St. Joseph (i.e., West Ressource, East Ressource, Benjamin, Fouquet) compared to the North Side. The above pattern of relative abundances among sites was apparent for both sampled skin locations, i.e. skin covering the gills and the back of the sharks (for details, see Table 4 a,b). Notably, putative core microbiome members together constituted a larger relative proportion of total bacterial sequences associated with black-tip reef shark skin off the outer St. Joseph Islands, i.e. East Ressource, Fouquet, and Benjamin, compared to West Ressource and North Side (Table 4 a, b). No OTU was significantly differentially abundant between the two locations of shark skin.

**Table 4:** Relative abundances (%) of putative core microbiome members of bacterial communities associated with visibly healthy and infected skin around the gills and visibly healthy skin on the back of black-tip reef sharks (*Carcharhinus melanopterus*) collected in the Amirante Islands (Seychelles), presented for **a)** gills and **b)** backs of sharks. Bacterial relative abundances are averaged within sites (data presented as means ± SD). Taxonomy: Numbers in brackets constitute bootstrap values; only bootstrap values < 100 are shown

Site						
	West Ressource	North Side	East Ressource	Fouquet	Benjamin	Taxonomy
a) Gills						
OTU00001	3.61 ± 2.95	2.86 ± 1.23	10.49 ± 3.76	8.61 ± 3.83	9.48 ± 5.54	f__*Rhodobacteraceae*_unclass.(86)
OTU00002	5.11 ± 4.62	1.76 ± 1.59	4.24 ± 3.75	8.28 ± 7.16	9.04 ± 2.72	g__*Alteromonas* unclass.
OTU00003	5.65 ± 1.87	1.21 ± 0.86	3.11 ± 1.50	2.53 ± 1.55	1.40 ± 1.20	f__*Pelagibacteraceae*_unclass.(99)
OTU00004	1.45 ± 1.54	0.09 ± 0.11	3.19 ± 1.28	4.38 ± 2.57	3.28 ± 0.63	o__Flavobacteriales_unclass.
OTU00005	2.02 ± 1.13	3.62 ± 1.74	1.22 ± 0.94	1.17 ± 0.50	1.3 ± 0.46	o__*Vibrionales*_unclass.(85)
OTU00006	2.07 ± 1.05	0.24 ± 0.14	2.08 ± 1.04	2.06 ± 1.07	1.62 ± 1.17	f__*OCS155*_unclass.
OTU00010	0.63 ± 0.70	0.07 ± 0.09	2.42 ± 1.11	2.02 ± 1.02	3.67 ± 1.96	o__*Oceanospirillales*_unclass.
OTU00011	0.8 ± 0.67	0.28 ± 0.35	1.69 ± 1.19	1.78 ± 0.74	0.98 ± 0.57	s__*Psychrobacter pacificensis*
OTU00014	0.98 ± 1.17	0.28 ± 0.33	2.13 ± 1.13	1.81 ± 0.80	0.96 ± 0.84	f__Flavobacteriaceae_unclass.
OTU00016	0.67 ± 0.72	0.78 ± 0.55	1.72 ± 0.96	1.42 ± 0.36	0.67 ± 0.074	s__*Pseudoalteromonas porphyrae*
OTU00019	1.04 ± 0.98	0.03 ± 0.03	1.57 ± 0.84	1.76 ± 0.89	1.09 ± 0.82	f__Halomonadaceae unclass.
Others	75.95 ± 4.83	88.78 ± 2.36	66.14 ± 8.33	64.17 ± 4.59	66.47 ± 8.04	
b) Back						
OTU00001	2.92 ± 2.39	2.55 ± 1.29	10.96 ± 4.01	8.33 ± 3.77	9.15 ± 4.74	f__Rhodobacteraceae_unclass.(86)
OTU00002	7.57 ± 7.78	2.88 ± 2.61	4.49 ± 2.29	8.14 ± 3.86	10.57 ± 6.65	g__*Alteromonas* unclass.
OTU00003	7.58 ± 5.39	1.36 ± 1.78	3.40 ± 1.37	7.77 ± 1.30	1.68 ± 1.26	f__Pelagibacteraceae_unclass.(99)
OTU00004	0.90 ± 0.61	0.08 ± 0.15	3.36 ± 1.37	8.31 ± 1.56	2.19 ± 0.82	o__Flavobacteriales_unclass.
OTU00005	1.49 ± 1.28	2.36 ± 0.90	1.81 ± 1.32	9.05 ± 0.42	1.76 ± 1.30	o__Vibrionales_unclass.(85)
OTU00006	2.41 ± 1.65	0.26 ± 0.28	2.62 ± 0.91	10.15 ± 1.06	1.96 ± 0.53	f__OCS155_unclass.
OTU00010	0.68 ± 0.91	0.04 ± 0.06	2.71 ± 1.10	11.44 ± 0.85	4.74 ± 3.34	o__Oceanospirillales_unclass.
OTU00011	1.00 ± 1.05	0.39 ± 0.48	3.42 ± 3.06	13.18 ± 0.83	1.64 ± 0.37	s__*Psychrobacter pacificensis*
OTU00014	0.88 ± 0.81	0.28 ± 0.34	2.15 ± 0.82	15.99 ± 0.89	1.28 ± 0.80	f__Flavobacteraceae_unclass.
OTU00016	0.70 ± 0.63	1.34 ± 1.02	2.08 ± 0.95	20.33 ± 0.45	0.78 ± 0.30	s__*Pseudalteromonas porphyrae*
OTU00019	1.08 ± 1.21	0.03 ± 0.05	1.89 ± 0.79	30.00 ± 1.03	1.50 ± 0.88	f__Halomonadaceae unclass.
Others	72.78 ± 7.39	88.43 ± 3.13	61.10 ± 7.76	58.56 ± 19.04	62.75 ± 4.52	

## Discussion

The present study investigated the bacterial skin microbiome of wild-caught black-tip reef sharks, *C. melanopterus*, from the Amirante Islands in the Seychelles, comparing visibly healthy individuals with individuals exhibiting tissue insult on the skin around the gills. High throughput 16S rRNA gene amplicon sequencing on the Illumina HiSeq platform revealed that the bacterial communities in those specimens with visibly healthy skin and those with insulted skin on the gills were statistically indistinguishable, i.e. bacterial community composition remained highly conserved upon tissue insult. Similarly, no differences were observed between samples from skin around the gills and from skin on the posterior back of the same sharks. Significant differences were only observed with respect to the sampling sites where the sharks were caught. The observed patterns align with our current understanding of black-tip reef shark ecology and the unique cutaneous structure of shark skin, suspected to hinder bacterial infection. Potential links between bacterial taxa and immune properties of shark skin should be addressed in future work, as discussed below.

### Bacterial community composition of black-tip reef shark skin

The bacterial community of black-tip reef shark skin investigated in the present study was comprised of a combination of several bacterial genera previously identified to be characteristic of shark skin [27], as well as bacterial taxa  common in a range of marine organisms and environments [40–43]. Bacteria previously reported characteristic of the thresher shark (*Alopias vulpinus*) skin microbiome, but absent in corresponding seawater samples were *Erythrobacter*, *Idiomarina*, *Marinobacter*, and *Pseudoalteromonas* [27]. Shotgun sequencing suggested these bacteria harbor potentially important functions, including the synthesis of photosynthate (*Erythrobacter*), heavy metal detoxification (*Idiomarina*), and lipopolysaccharide degradation (*Marinobacter*), the latter of which may mediate and reduce host inflammatory responses [27, 44]. Several *Pseudoalteromonas* species produce compounds with bioactivity against prokaryotes and eukaryotes, affecting biofilm formation and biofouling [45, 46]. While these bacteria are metabolically diverse and may exhibit different metabolic traits even at the strain level, they may have a potentially critical role in structuring the shark skin microbiome and aid in the prevention of bacterial infection of (injured) skin. Notably these four bacterial genera occur on both thresher shark and black-tip reef shark skin – two species of shark exhibiting very different ecological niches and lifestyles [47] – suggesting a potentially conserved role in shark skin health.

We identified eleven core microbiome members of black-tip reef shark skin. Two of these could be annotated to the species level: OTU11 *Psychrobacter pacificensis* and OTU16 *Pseudoalteromonas porphyrae*. *Psychrobacters* were previously identified as core microbiome members of humpback whale skin and have been linked with whale health and immunity [48, 49]. Notably, *Psychrobacters* occur in the skin mucus of bony fish [50] and pure isolates have shown inhibition to aquatic fungal pathogens [51]. The presence of *Psychrobacters* on the skin of whale [48], shark [27 and in the present study], and bony fish suggests *Psychrobacters* may be ubiquitous and functionally important skin microbiota of aquatic vertebrates. While it should be noted that the identification of the core microbiome is always only an approximation, biased by sample design and sample size, arguably the present study features a reasonable number of samples covering a fairly comprehensive study area. This is further supported by the identification of *Pseudoalteromonas* and *Psychrobacter* as core microbiome members of black-tip reef shark skin, given the contemporary literature (see above). In this regard, future work should include the isolation of bacteria to assess their potential contribution to shark skin health. In particular, targeting the production and activity of antibiotics, antimicrobial peptides, and other bioactive compounds may provide clues as to the importance of bacteria.

In the present study, the bacterial communities of shark skin were conserved with regard to skin health state and sampled skin location, but exhibited differences between sampling locations within the Amirante Islands. While the sites are only a few kilometers away from each other, relative abundances of core microbiome members (Table 4) likely reflect oceanographic connectivity and movement of sharks between the three St. Joseph islands, i.e. East Ressource, Fouquet, and Benjamin, as opposed to the other two sites, North Side (off d’Arros) and West Ressource (belonging to the St. Joseph reef group, but situated closer to d’Arros). Thereby, the shark skin microbiome may be reflecting seawater properties, connectivity, and potentially anthropogenic impact of the respective sampling locations within the study area, while transmission of surface microbes between individual sharks using a reef area may also be a factor, since this species often feed in close proximity to one another. This observed location-specific pattern is in line with our understanding of the movement ecology of the black-tip reef shark, since the species exhibits the smallest known home range within the genus *Carcharhinus*, in some cases being known to not (or rarely) cross between adjacent habitats separated by channels of as little as 1.7 km [52–54]. Indeed, an acoustic tagging study undertaken in parallel at the same locations as the present study has shown that in contrast to other species, black-tip reef sharks rarely cross the deeper water between D’Arros and St. Joseph island [55], likely due to the risk of predation by larger shark species [56, 57]. The distances between the islands off St. Joseph reef (East Ressource, Fouquet, Benjamin) however are well within the home ranges reported for black-tip reef sharks, and cross-reef migration in this area has been observed [55]. The same may apply to the sites North Side and West Ressource. Hence, between-island movement of sharks likely explains observed patterns in skin-associated bacterial communities in the present study.

### Potential causes of skin insults in black-tip reef sharks

The bacterial community composition conserved in both visibly healthy and insulted skin covering the gill area strongly suggests that despite sometimes extensive visible skin injury, there is no indication of severe bacterial infection as characterized by the propagation of opportunistic or pathogenic bacteria. Indeed, not every wound progresses to being infected, and, even when inflammation is present, bacterial infection may not occur [61]. While the skin insult might have been caused by infection with fungi [17] or monogenean worms [16, 23], skin-associated bacteria likely would have exhibited a ‘secondary’ change in community composition in response to primary eukaryotic infection. Therefore, eukaryotic infection as the cause of skin insults may be unlikely. Rather, skin insults observed in the black-tip reef shark samples may have been a consequence of mechanic disruption of the skin. Due to the limitations of vessel-based field work, we could not directly observe the cause of skin insults, or track the development of skin insults over time, but as the behavior of black-tip reef sharks is reasonably well understood, it is conceivable to interpret the insults as the result of inter- and intraspecific antagonistic interactions. In some cases, this could have occurred during the mating act, in which male sharks commonly injure females during courtship and intromission by biting on to one of their pectoral fins and gill area, or when entangled both partners may come into physical contact with nearby rocks or coral [54]. However, similar skin insults were observed in both female and male sharks, the two sexes exhibiting similar patterns of damage, being concentrated on the anterior flank, immediately around the gills. While this might be suggestive of damage inflicted by a gill parasite, none were evident on quick inspection in the field. Other causes of mechanical disruption of the skin in black-tip reef sharks are also possible, such as boat strike, or intraspecific aggressive behavior or predation attempts by larger sharks [15, 56], although most injuries did not suggest these causes in the present study.

### Conserved bacterial communities on healthy and insulted skin: structural properties of shark skin and immune responses

Skin acts as a physical barrier to the surrounding environment, protecting against invasion by foreign substances and pathogens [26, 30]. Skin microbiomes are shaped in part by properties, such as topographical location, endogenous host factors, and exogenous environmental factors [27, 28, 58]. Skin insults, including injury, lesions, inflammation, infection, or disease, are commonly associated with microbiome shifts [31–33, 35]. Whether or not progression from bacterial colonization to infection occurs depends first and foremost on the host’s immune response [61]. In the present study, bacterial community composition and structure was highly conserved between healthy and insulted shark skin samples based on 16S rRNA gene amplicon sequencing. From the bacterial community profiles, any progression from bacterial colonization to severe infection (characterized by the propagation of potential pathogens) was notably absent, even though a mild bacterial infection may have gone undetected.

It is important to acknowledge that bacterial community profiles based on 16S rRNA gene amplicon sequencing alone cannot address mechanisms underlying the conserved bacterial community composition in visibly healthy and insulted shark skin. Nonetheless, the present study provides insight into the ecology of shark skin microbiomes and highlights that mechanistic studies will be required for a better understanding of bacterial infection and immunity in sharks. Specifically, future studies should target whether shark skin and its associated bacteria are able to maintain skin functioning under environmental stress or severe tissue insult, as previously suggested [27, 38], and whether this is linked to endogenous host factors.

Endogenous host factors encompass physical properties of the skin, such as its microtexture [59, 60] and cutaneous immune response repertoires, which may modulate skin-associated bacterial communities [28]. In sharks, skin microtexture potentially constitutes an important host factor that contributes to the structuring of bacterial communities. As described previously, shark skin exhibits a unique cutaneous structure, morphologically setting it apart from the skin of bony fish. Specifically, shark skin is characterized by dermal denticles, which protrude through both the epidermis and mucus layer. This results in a textured surface with pronounced microscopic ridging, which appears to greatly reduce microbial settlement [59, 60] and which has likewise been found to reduce microbial settlement on a similarly textured experimental substrate [68]. Another potential factor mediating skin bacterial communities in black-tip reef sharks may be the production of antimicrobial compounds resident in the skin or skin mucus layer. While the presence of such compounds has been previously reported from other sharks (e.g., squalamines, a group of water-soluble antibiotics associated with shark organs and tissues) and from bony fish [62–64], their role in countering bacterial infection *in hospite* still needs to be assessed. Hence, the potential role of resident bacterial members in structuring the shark skin microbiome [30, 45] and supporting wound healing by mediating the inflammatory response [27, 44, 65, 66] should be a focus of future research efforts. Finally, as in all cartilaginous fish, the shark immune system encompasses adaptive components (e.g., an immunoglobulin system) and appears to be capable of immunological recall [67]. If and how the adaptive immune system plays into the significant capacity for wound healing in the black-tip reef shark [57] remains yet to be determined. Nevertheless, our finding of conserved bacterial community structures between healthy and injured black tip reef shark skin highlights the putative immense capacity to thwart bacterial infection and support rapid wound healing.

## Conclusions

The present study employed high throughput 16S rRNA gene amplicon sequencing to characterize skin-associated bacterial communities of black-tip reef sharks from the Amirante Islands in the Seychelles. Comparison of visibly healthy and insulted skin samples from the gill areas, as well as healthy skin samples from the back of the sharks, showed no differences in bacterial community composition, suggesting conservation of microbiome structure even under injury. At present the relative contribution of animal host factors, such as the ultrastructure of the shark skin to limit bacterial settlement or factors attributable to  the resident bacterial community, such as the production of antimicrobial compounds, is unknown. Both factors may help select and preserve the native bacterial community even upon tissue insult and may likewise counter infection. In contrast to the similarities between healthy and injured skin samples, differences related to collection sites suggest that bacterial community structure may respond to exogenous environmental factors. For a better understanding of the roles and properties of resident bacteria of shark skin, future studies should aim for a comprehensive approach combining bacterial community profiling with host immune assays and screening for bioactive compounds from bacterial isolates. Such a combined approach may help elucidate the mechanisms underlying the considerable capacity for wound healing and microbiome resilience prevalent in sharks.

## Methods

### Sampling sites, shark sampling, and swab collection

Black-tip reef sharks were wild-caught and sampled in the Amirante Islands, Seychelles, from 27 March – 19 April 2017 (Fig. 1a; Additional file 6: Table S3). Sampling locations included St. Joseph Atoll (Four Sites: Western Ressource, Eastern Ressource, Fouquet, and Benjamin) and D’Arros Island (North Site; Fig. 1a). Overall, the sites are located a few hundred meters (within the St. Joseph Island group) to a few kilometers away from each other (between North Site off D’Arros and the St. Joseph island group). Notably, Ressource is located about halfway between D’Arros (in the West) and St. Joseph (in the East), however its western reefs are facing D’Arros, and its eastern reefs are facing the St. Joseph island group. Likely, W. and E. Ressource are therefore more strongly oceanographically connected to D’Arros and St. Joseph, respectively.

A total of 44 black-tip reef sharks were caught alive by circle hook and line; the sharks remained partially submersed at the side of the boat during sampling and were then released unharmed. Skin sections from which mucus swab samples were taken were briefly exposed to air during the sampling. For each shark, the left side of the body was sampled. Specifically, one sample was taken from the skin covering and around the gill area, and a second sample from the skin on the back just below the first dorsal fin, by swabbing the surface with individual forceps-held sterile cotton swabs (Nuova Aptaca, Italy) so as to collect a sample of the mucus. Overall, 44 mucus swabs were collected from each of (a) the skin covering and around the gills (‘gills’) and (b) the dorsal part of the flank (‘back’), resulting in 88 swab samples in total. Swabs were selected as a means of non-invasive sampling [69]. Swab samples were immediately transferred into RNAlater and stored at 5 °C and subsequently − 20 °C until further processing. Sampling the same shark twice was avoided by taking pictures of each side of the first dorsal fin to document individual markings on each shark, an approach which is commonly used for identification of individuals. In addition, all sharks sampled were marked by removing the extreme tip of the anal fin.

For each sampled shark, health condition (‘healthy’ and ‘insulted’) of the skin covering gills was recorded. ‘Healthy’ shark samples did not exhibit any visible signs of tissue insult on the skin surrounding the gill area. ‘Insulted’ shark samples exhibited marked tissue insult (Fig. 1c). None of the sharks exhibited any visible skin insults on the ‘back’ area, i.e., in the dorsal part of the flank. Sampling of insulted skin area entailed sampling directly across the insulted area on the skin covering the gills in order to determine whether bacterial community composition was different in insulted skin areas compared to visibly healthy skin. Due to practical considerations, time constraints, and the fact that observation of shark matings are very rare, we were not able to observe when individual skin insults were inflicted, nor to track the development of insults over time. Hence, the age of skin insults at the time of sampling is unknown.

### DNA extraction, PCR conditions, sequencing library preparation

Prior to DNA extraction, swabs were thawed at room temperature, removed from RNAlater solution, each placed in a sterile 1.5 ml Eppendorf tube, and air-dried for 10 min. DNA extraction was conducted using a modified ‘Wayne’s’ protocol [70]. 375 μl of freshly prepared extraction buffer (100 mM Tris, 100 mM EDTA, 100 mM NaCl, 1% SDS) was added to each tube. Samples were vortexed and incubated at 65 °C for 2 h. 1 μl of RNase A was added 15 min before the end of the incubation. After the incubation samples were vortexed again, the swab removed, and the sample put on ice. 94 μl of 5 M KOAc was added to each tube, vortexed, and incubated on ice for 10 min. Samples were then centrifuged for 10 min (14,000 rpm, RT). The supernatant was transferred to a new tube and 300 μL of 100% isopropanol added, mixed gently, and incubated for 5 min at RT. Samples were then spun at maximum speed at RT for 20 min. The supernatant was discarded by pipetting. 150 μl of 70% ethanol were added to each tube, mixed gently, and then tubes were centrifuged at maximum speed for 10 min. The resulting DNA pellet was air-dried for 15 min and subsequently resuspended overnight at 4 °C in 20 μl of 0.1 M Tris. Isolated DNA was quantified on the NanoDrop 2000C spectrophotometer (Themo Fisher Scientific, USA). In addition to DNA extractions from samples, mock DNA extractions (no sample, reagents only) were conducted.

For all samples, PCR amplifications were performed in triplicates using Qiagen Multiplex PCR Kit (Qiagen, Germany) with primers containing Illumina adapters (underlined below). For the 16S rRNA gene sequencing, we amplified the hypervariable regions V5 and V6 of the bacterial 16S rRNA gene. Primers 16SMiSeqF-Andersson 5′TCGTCGGCAGCGTCAGATGTGTATAAGAGACAGAGGATTAGATACCCTGGTA-3′ and 16SMiSeqR-Andersson 5′-GTCTCGTGGGCTCGGAGATGTGTATAAGAGACAGCRRCACGAGCTGACGAC-3′ were used, which have previously been shown to amplify well with marine templates [41, 71]. Individual PCRs were run using 5 μl Qiagen Mix, 0.2 μl of each 10 μM primer mix, 1 μl of DNA template, and RNase-free water to adjust to a final reaction volume of 10 μl. In addition to samples, PCRs were run for templates from the mock DNA extraction, along with mock PCRs (no template input). Thermal cycling conditions for 16S rRNA gene PCRs were: 95 °C for 15 min, followed by 27 cycles of 95 °C for 30 s, 55 °C for 90 s, 72 °C for 30 s, and a final extension cycle of 72 °C at 10 min. Five µl of each PCR product were run on an 1% agarose gel to visualize successful amplification. Sample triplicates were subsequently pooled and then purified with Illustra ExoProStar 1-Step (GE Healthcare Life Sciences, UK). Purified PCR products were subjected to an indexing PCR (8 cycles) to add Nextera XT indexing and sequencing adapters (Illumina, USA) according to the manufacturer’s protocol. Indexed products were again purified and normalized with the SequalPrep Normalization Plate Kit (Thermo Fisher Scientific, USA), followed by quantification on the BioAnalyzer (Agilent Technologies, USA) and QuBit (Quant-IT dsDNA High Sensitivity Assay Kit; Invitrogen, USA), and pooled in equimolar ratios. The library was sequenced at 15 pM with 2% phiX on the Illumina HiSeq 2500, 2 × 250 bp end, Rapid run, 500 cycles, according to the manufacturer’s specifications at the Bioscience Core Lab (BCL) at the King Abdullah University of Science and Technology (KAUST), Saudi Arabia. Libraries sequenced included samples along with PCR products from mock DNA extractions and mock PCRs as a negative control to account for environmental and laboratory contamination.

### Sequencing data analysis

To assess bacterial community composition of shark skin of different health states and from different locations on shark skin, we sequenced 88 16S rRNA gene amplicon libraries (44 gill + 44 back samples, distributed over 28 visibly healthy + 16 injured specimens (Additional file 4: Table S1). Bacterial 16S rRNA gene amplicon sequences were processed using *mothur* version 1.39.0 using the *mothur* MiSeq SOP (accession date: May 2018; [72] (Additional file 1: Methods S1). In brief, sequences were assembled into contigs and quality trimmed. Identical sequences (duplicates) were merged. Singletons and rare sequences (*n* < 10 over all samples) were removed. This resulted in 18,022,131 sequences distributed over 88 shark samples [44 gill and 44 back skin samples; distributed over 28 visibly healthy and 16 infected individuals]. After trimming, 14,320,306 sequences with average length of 292 bp remained. Remaining sequences were aligned against the SILVA database (release 119; [73]) and pre-clustered (2 bp difference; [74]). Chimeric sequences were removed using the VSEARCH command [75]. Unwanted sequences assigned to chloroplasts, mitochondria, archaea, and eukaryotes were removed, clustered into Operational Taxonomic Units (OTUs, 97% similarity cutoff), and annotated against the Greengenes database (release gg_13_8_99, [76]). Notably, the here-used primer pair 784F-1016R is not well suited for the amplification of archaeal 16S rRNA gene sequences, as assessed using the TestPrime tool in SILVA (https://www.arb-silva.de/search/testprime/): coverage and specificity of this primer pair against the SILVA database was 0 for archaea. For this reason, any sequences assigned as archaea were removed during the remove.lineages step in *mothur* (for details, please refer to Additional file 1: Methods S1). After removal of these unwanted sequences 10,674,925 sequences were retained. Subsequently, sequences were subsampled to 24,190 sequences per sample, and low abundance taxa (< 10 sequences across all samples) were removed. Environmental and laboratory contaminants were removed based on sequencing results of mock extractions and mock PCRs (*Staphylococcus* OTU 00008, *Propionibacterium* OTU00024, *Caulobacter* OTU00099, *Pelomonas* OTU00148, *Sphingomonas* OTU00196, *Brevibacterium* OTU00238, *Sediminibacterium* OTU00290, *Corynebacterium* OTU00333, *Aquabacterium* OTU00511, *Microbispora* OTU00598, *Bosea* OTU00601, *Delftia* OTU00745, *Rubricoccus* OTU00949, *Polyangiaceae* sp. OTU01000 and OTU02727, *Saprospiraceae* sp. OTU01314, *Myroides* OTU02959, and *Frankiaceae* OTU04398, some of which are common lab or kit contaminants [77], along with *Endozoicomonas* OTUs 00022, 00065, 00121, 00301, a marine bacterium maintained in permanent culture in the processing lab). After removal of sequences related to contaminants, a total of 2,034,047 sequences (on average 23,114 sequences per sample) were retained for subsequent analyses. Alpha diversity metrices were calculated with the *summary.single* command as implemented in *mothur* [78]. The bacterial ‘core’ microbiome was extracted with the *get*.*coremicrobiome* command as implemented in *mothur* at an 80 and 50% cutoff (i.e., present in at least 80 and 50% of all samples, respectively) [78]. All raw sequence data are accessible under NCBI’s BioProject PRJNA498626.

### Statistical analysis

Sequence counts of the OTU abundance table were converted into relative abundance data, normalized, and square root transformed. Bray-Curtis similarity was applied on the square root transformed data [79]. Subsequently, permutational multivariate analysis of variance (PERMANOVA [80]) was conducted. To assess differences in bacterial community composition between sharks with visibly healthy and insulted skin covering the gill area, PERMANOVAs were run separately on samples from gills and back using *adonis* [80]. To assess differences in bacterial community composition for sampling sites in the Amirante Islands, ‘site’ was assigned a fixed factor and shark ‘sex’ was assigned a random factor nested in ‘site’. Subsequently, 9999 permutations of residuals under a reduced model were conducted based on Bray–Curtis distances between root transformed samples. In addition, pairwise Analysis of Similarity (ANOSIM) comparisons with 9999 permutations were run for factor sampling site (‘site’) to assess which sites were significantly different from each other. Beta diversity differences for bacterial community composition were visualized in a principal coordinate analysis based on a Bray-Curtis dissimilarity matrix. A two-way ANOVA run in R [81] revealed the main contributing bacterial families responsible for differences regarding shark health state and sampling site.

## Additional files

Additional file 1: **Methods S1** mothur script for 16S rRNA gene amplicon profiling of shark skin-associated bacterial communities. (TXT 15 kb)

Additional file 2: **Figure S1** Representative rarefaction curves for bacterial community sequencing efforts of healthy and insulted skin samples of black-tip reef sharks (*Carcharhinus melanopterus*) collected at five sites in the Amirante Islands, Seychelles. To facilitate presentation, one representative sample of a healthy and insulted skin sample is provided for each of the five sites, except for the site Benjamin, where no visibly healthy sharks could be sampled. **a)** Samples from skin around the gills, **b)** samples from skin on the back. Plateauing curves suggest adequate sequencing effort. (DOCX 704 kb)

Additional file 3: **Figure S2** Family-level stacked bar plots for bacterial community composition of healthy and insulted skin samples of black-tip reef sharks (*Carcharhinus melanopterus*) collected at different sites in the Amirante Islands, Seychelles. Each bar represents an individual sample. **a)** Samples from the skin around the gill area. **b)** Samples from the skin on the back of the shark. (DOCX 350 kb)

Additional file 4: **Table S1** OTU abundance table showing the distribution of bacterial 16S rRNA gene sequences for each OTU over samples. Bacterial communities were associated with visibly healthy and infected skin around the gills and visibly healthy skin on the back of black-tip reef sharks (*Carcharhinus melanopterus*) collected in the Amirante Islands (Seychelles). (TXT 3913 kb)

Additional file 5: **Table S2** Detailed results of two-way ANOVA with subsequent FDR correction to test for differentially abundant bacterial OTUs of black-tip reef sharks (*Carcharhinus melanopterus*) between shark collection sites, location of shark skin tissue, and the interaction of both. (TXT 620 kb)

Additional file 6: **Table S3** Details on collection date, location, health state, and sex of individual blacktip reef sharks collected in 2017 around the Amirante Islands, Seychelles. Shark identifiers printed in bold indicate individuals with observed skin insult. (DOCX 19 kb)

## Data Availability

Sequence data determined in this study are available under NCBI BioProject ID PRJNA498626 (https://www.ncbi.nlm.nih.gov/bioproject/PRJNA498626). Abundant shark skin bacterial microbiome OTU reference sequences are available under GenBank Accession numbers MK577282 - MK577302 (https://www.ncbi.nlm.nih.gov/nuccore/?term=MK577282:MK577302[accn]).

## Abbreviations

ANOSIM: Analysis of Similarity
ANOVA: Analysis of Variance
bp: Base pair
DNA: Desoxyribonucleic acid
FDR: False discovery rate
MS: Mean of squares
NCBI: National Center for Biotechnology Information
OTU: Operational taxonomic unit
PCoA: Principal Coordinate Analysis
PCR: Polymerase Chain Reaction
PERMANOVA: Permutational Analysis of Variance
rpm: Rotations per minute
RT: Room temperature
SE: Standard error
SOP: Standard operation procedure
SS: Sum of squares

## References

[1] Baum JK, Myers RA (2004). Shifting baselines and the decline of pelagic sharks in the Gulf of Mexico. Ecol Lett.

[2] Baum JK, Myers RA, Kehler DG, Worm B, Harley SJ, Doherty PA (2003). Collapse and conservation of shark populations in the Northwest Atlantic. Science..

[3] Robbins WD, Hisano M, Connolly SR, Choat JH (2006). Ongoing collapse of coral-reef shark populations. Curr Biol.

[4] Ferretti F, Myers RA, Serena F, Lotze HK (2008). Loss of large predatory sharks from the Mediterranean sea. Conserv Biol.

[5] Graham NAJ, Spalding MD, Sheppard CRC (2010). Reef shark declines in remote atolls highlight the need for multi-faceted conservation action. Aquat Conserv Mar Freshwat Ecosyst.

[6] Dulvy NK, Baum JK, Clarke S, Compagno LIV, Cortes E, Al E (2008). You can swim but you can’t hide: the global status and conservation of oceanic pelagic sharks and rays. Conserv Mar Freshw Ecosyst.

[7] IUCN (2016). UCN red list of threatened species. version 4, update 2016–3.

[8] Ormond R, Gore M, Bladon A, Dubock O, Kohler J, Millar C (2016). Protecting Cayman Island sharks : monitoring, movement and motive. Proceedings of the 69th Gulf and Caribbean Fisheries Institute.

[9] Myers RA, Baum JK, Shepherd TD, Powers SP, Peterson CH (2007). Cascading effects of the loss of apex predatory sharks from a coastal ocean. Science..

[10] Ward-Paige CA (2017). A global overview of shark sanctuary regulations and their impact on shark fisheries. Mar Policy.

[11] Ward JR, Lafferty KD (2004). The elusive baseline of marine disease: are diseases in ocean ecosystems increasing?. PLoS Biol.

[12] Ostrander GK, Cheng KC, Wolf JC, Wolfe MJ (2004). Shark cartilage, cancer and the growing threat of pseudoscience. Cancer Res.

[13] Dagleish MP, Baily JL, Foster G, Reid RJ, Barley J (2010). The first report of disease in a basking shark (*Cetorhinus maximus*). J Comp Pathol.

[14] Reif W (1978). Wound healing in sharks. Zoomorphologie..

[15] Chin A, Mourier J, Rummer JL (2015). Blacktip reef sharks (*Carcharhinus melanopterus*) show high capacity for wound healing and recovery following injury. Conserv Physiol.

[16] Cheung PJ, Nigrelli RF, Ruggieri GD, Osborn AC (1982). Treatment of skin lesions in captive lemon sharks, *Negaprion brevirostris* (Poey), caused by monogeneans (*Devmophthirius* sp.). J Fish Dis.

[17] Crow GL, Brock JA, Kaiser S, Crow GL, Brock JA, Kaiser S (1995). *Fusarium solani* fungal infection of the lateral line canal system in captive scalloped hammerhead sharks (*Sphyma lewini*) in Hawaii. J Wildl Dis.

[18] Grimes DJ, Burgess J, Crunkleton JA, Brayton PR, Colwell RR (1989). Potential invasive factors associated with *Vibrio carchariae*, an opportunistic pathogen for sharks. J Fish Dis.

[19] Grimes DJ, Gruber SH, May EB (1985). Experimental infection of lemon sharks, *Negaprion brevirostris* (Poey), with *Vibrio* species. J Fish Dis.

[20] Grimes DJ, Colwell RR, Stemmler J, Hada H, Maneval D, Hetrick FM (1984). *Vibrio* species as agents of elasmobranch disease. Helgolaender Meeresuntersuchungen.

[21] Muhvich AG, Reimschuessel R, Lipsky MM, Bennett RO (1989). *Fusarium solani* isolated from newborn bonnethead sharks, *Sphyrna tiburo* (L.). J Fish Dis.

[22] Poynton SL, Campbell TW, Palm HW (1997). Skin lesions in captive lemon sharks *Negaprion brevirostris* (Carcharhinidae) associated with the monogenean *Neodermophthirius harkemai*. Dis Aquat Org.

[23] Bullard Stephen A., Frasca Salvatore, Benz George W. (2000). Skin Lesions Caused byDermophthirius penneri(Monogenea: Microbothriidae) on Wild-Caught Blacktip Sharks (Carcharhinus limbatus). Journal of Parasitology.

[24] Bang C, Dagan T, Deines P, Dubilier N, Duschl WJ, Fraune S (2018). Metaorganisms in extreme environments: do microbes play a role in organismal adaptation?. Zoology..

[25] McFall-Ngai M, Hadfield MG, Bosch TCG, Carey HV, Domazet-Lošo T, Douglas AE (2013). Animals in a bacterial world, a new imperative for the life sciences. Proc Natl Acad Sci.

[26] Rohwer F, Seguritan V, Azam F, Knowlton N (2002). Diversity and distribution of coral-associated bacteria. Mar Ecol Prog Ser.

[27] Doane MP, Haggerty JM (2017). The skin microbiome of the common thresher shark (*Alopias vulpinus)* has low taxonomic and gene function beta-diversity. Environ Microbiol.

[28] Grice EA, Segre JA (2011). The skin microbiome. Nat Rev Microbiol.

[29] Egan S, Gardiner M (2016). Microbial dysbiosis: rethinking disease in marine ecosystems. Front Microbiol.

[30] Nakatsuji T, Chen TH, Narala S, Chun KA, Two AM, Yun T (2017). Antimicrobials from human skin commensal bacteria protect against *Staphylococcus aureus* and are deficient in atopic dermatitis. Sci Transl Med.

[31] Cárdenas A, Rodriguez-R LM, Pizarro V, Cadavid LF, Arévalo-Ferro C, Cardenas A (2012). Shifts in bacterial communities of two Caribbean reef-building coral species affected by white plague disease. ISME J.

[32] Kong H. H., Oh J., Deming C., Conlan S., Grice E. A., Beatson M. A., Nomicos E., Polley E. C., Komarow H. D., Murray P. R., Turner M. L., Segre J. A. (2012). Temporal shifts in the skin microbiome associated with disease flares and treatment in children with atopic dermatitis. Genome Research.

[33] Roder C, Arif C, Daniels C, Weil E, Voolstra CR (2014). Bacterial profiling of white plague disease across corals and oceans indicates a conserved and distinct disease microbiome. Mol Ecol.

[34] Gignoux-Wolfsohn SA, Aronson FM, Vollmer SV. Complex interactions between potentially pathogenic, opportunistic, and resident bacteria emerge during infection on a reef-building coral. FEMS Microbiol Ecol. 2017;93:fix080. 10.1093/femsec/fix080 28637338

[35] Shotts EB, Albert TF, Wooley RE, Brown J (1990). Microflora associated with the skin of the bowhead whale (*Balaena mysticetus*). J Wildl Dis.

[36] Becker MH, Harris RN (2010). Cutaneous bacteria of the redback salamander prevent morbidity associated with a lethal disease. PLoS One.

[37] Zaneveld J, McMinds R, Vega Thurber RL (2017). Stress and stability: applying the Anna Karenina principle to animal microbiomes. Nat Microbiol.

[38] Reid G, Younes JA, Van der Mei HC, Gloor GB, Knight R, Busscher HJ (2011). Microbiota restoration: natural and supplemented recovery of human microbial communities. Nat Rev Microbiol.

[39] Vignaud T, Mourier J, Maynard J, Leblois R, Spaet J, Clua E (2014). Blacktip reef sharks, *Carcharhinus melanopterus*, have high genetic structure and varying demographic histories in their Indo-Pacific range. Mol Ecol.

[40] Pfreundt U, Spungin D, Bonnet S, Berman-frank I, Hess WR (2016). Global analysis of gene expression dynamics within the marine microbial community during the VAHINE mesocosm experiment in the Southwest Pacific. Biogeosciences..

[41] Pogoreutz C, Rädecker N, Cárdenas A, Gärdes A, Wild C, Voolstra CR. Dominance of *Endozoicomonas* bacteria throughout coral bleaching and mortality suggests structural inflexibility of the *Pocillopora verrucosa* microbiome. Ecol Evol. 2018;8:2240–52. 10.1002/ece3.3830 PMC5817147 29468040

[42] Raina JB, Tapiolas D, Willis BL, Bourne DG (2009). Coral-associated bacteria and their role in the biogeochemical cycling of sulfur. Appl Environ Microbiol.

[43] Romanenko LA, Schumann P, Rohde M, Mikhailov VV, Stackebrandt E (2002). *Halomonas halocynthiae* sp. nov., isolated from the marine ascidian *Halocynthia aurantium*. Int J Syst Evol Microbiol.

[44] Kuepper FC, Gaquerel E, Boneberg EM, Morath S, Salauen JP, Potin P (2006). Early events in the perception of lipopolysaccharides in the brown alga *Laminaria digitata* include an oxidative burst and activation of fatty acid oxidation cascades. J Exp Bot.

[45] Bowman JP (2007). Bioactive compound synthetic capacity and ecological significance of marine bacterial genus *Pseudoalteromonas*. Mar Drugs.

[46] Ballestriero F, Thomas T, Burke C, Egan S, Kjelleberg S (2010). Identification of compounds with bioactivity against the nematode *Caenorhabditis elegans* by a screen based on the functional genomics of the marine bacterium *Pseudoalteromonas tunicata* D2. Appl Environ Microbiol.

[47] Compagno LIV (2002). Sharks of the world.

[48] Apprill A, Robbins J, Eren AM, Pack AA, Reveillaud J, Mattila D (2014). Humpback whale populations share a core skin bacterial community: towards a health index for marine mammals?. PLoS One.

[49] Bierlich KC, Miller C, DeForce E, Friedlaender AS, Johnston DW, Apprill A (2017). Temporal and regional variability in the skin microbiome of humpback whales along the Western Antarctic Peninsula. Appl Environ Microbiol.

[50] Juni E (1992). The genus *Psychrobacter*. The prokaryotes.

[51] Lowrey L, Woodhams DC, Tacchi L, Salinas I. Topographical mapping of the rainbow trout (*Oncorhynchus mykiss*) microbiome reveals a diverse bacterial community in the skin with antifungal properties. Appl Environ Microbiol. 2015;81:01826. 10.1128/AEM.01826-15 PMC4561705 26209676

[52] Papastamatiou YPP, Lwe CGL, Caselle JEC, Friedlander A (2009). Scale-dependent effects of habitat on movements and path structure of reef sharks at a predator-dominated atoll. Ecology..

[53] Mourier Johann, Vercelloni Julie, Planes Serge (2012). Evidence of social communities in a spatially structured network of a free-ranging shark species. Animal Behaviour.

[54] McCauley D, Papastamatiou Y, Young H (2010). An observation of mating in free-ranging blacktip reef sharks, *Carcharhinus melanopterus*. Pacific Biosci.

[55] Lea JS, Humphries NE, von Brandis RG, Clarke CR, Sims DW (2016). Acoustic telemetry and network analysis reveal the space use of multiple reef predators and enhance marine protected area design. Proc R Soc B Biol Sci.

[56] Mourier J, Planes S, Buray N (2013). Trophic interactions at the top of the coral reef food chain. Coral Reefs.

[57] Chin Andrew, Mourier Johann, Rummer Jodie L. (2015). Blacktip reef sharks (Carcharhinus melanopterus) show high capacity for wound healing and recovery following injury. Conservation Physiology.

[58] Fierer N., Hamady M., Lauber C. L., Knight R. (2008). The influence of sex, handedness, and washing on the diversity of hand surface bacteria. Proceedings of the National Academy of Sciences.

[59] Sullivan T, Regan F (2011). The characterization, replication and testing of dermal denticles of *Scyliorhinus canicula* for physical mechanisms of biofouling prevention. Bioinspired Bomim.

[60] Reddy ST, Chung KK, McDaniel CJ, Darouiche RO, Landman J, Brennan AB (2011). Micropatterned surfaces for reducing the risk of catheter-associated urinary tract infection: an *in vitro* study on the effect of sharklet micropatterned surfaces to inhibit bacterial colonization and migration of uropathogenic *Escherichia coli*. J Endourol.

[61] Edwards R, Harding KG (2004). Bacteria and wound healing. Curr Opin Infect Dis.

[62] Moore KS, Wehrlit S, Roder H, Rogers M, Forrest JN, McCrimmon D (1993). Squalamine : An aminosterol antibiotic from the shark. Proc Natl Acad Sci.

[63] Shinnar AE, Allen E, Peck A, Tal L, Goldstein R, Roberts L. Squalamine family of aminosterol antibiotics from various shark species. FASEB J. 2007:A1000.

[64] Esteban MA. An overview of the immunological defenses in fish skin. ISRN Immunol. 2012;4:116–128.

[65] Lai Yuping, Di Nardo Anna, Nakatsuji Teruaki, Leichtle Anke, Yang Yan, Cogen Anna L, Wu Zi-Rong, Hooper Lora V, Schmidt Richard R, von Aulock Sonja, Radek Katherine A, Huang Chun-Ming, Ryan Allen F, Gallo Richard L (2009). Commensal bacteria regulate Toll-like receptor 3–dependent inflammation after skin injury. Nature Medicine.

[66] Pasparakis M, Haase I, Nestle FO (2014). Mechanisms regulating skin immunity and inflammation. Nat Rev Immunol.

[67] Dooley H, Flajnik MF. Antibody repertoire development in cartilaginous fish. Developmental & Comparative Immunology. 2006;30:43–56. 10.1016/j.dci.2005.06.022 16146649

[68] Mann EE, Manna D, Mettetal MR, May RM, Dannemiller EM, Chung KK (2014). Surface micropattern limits bacterial contamination. Antimicrob Resist Infect Control.

[69] Kittelmann Sandra, Kirk Michelle R., Jonker Arjan, McCulloch Alan, Janssen Peter H. (2015). Buccal Swabbing as a Noninvasive Method To Determine Bacterial, Archaeal, and Eukaryotic Microbial Community Structures in the Rumen. Applied and Environmental Microbiology.

[70] Wilson K, Li Y, Whan V, Lehnert S, Byrne K, Moore S (2002). Genetic mapping of the black tiger shrimp *Penaeus monodon* with amplified fragment length polymorphism. Aquaculture..

[71] Bayer T, Neave MJ, Alsheikh-Hussain A, Aranda M, Yum LK, Mincer T (2013). The microbiome of the Red Sea coral *Stylophora pistillata* is dominated by tissue-associated *Endozoicomonas* bacteria. Appl Environ Microbiol.

[72] Schloss PD, Westcott SL, Ryabin T, Hall JR, Hartmann M, Hollister EB (2009). Introducing mothur: open-source, platform-independent, community-supported software for describing and comparing microbial communities. Appl Environ Microbiol.

[73] Pruesse E, Quast C, Knittel K, Fuchs BM, Ludwig W, Peplies J (2007). SILVA: a comprehensive online resource for quality checked and aligned ribosomal RNA sequence data compatible with ARB. Nucleic Acids Res.

[74] Huse SM, Welch DM, Morrison HG, Sogin ML (2010). Ironing out the wrinkles in the rare biosphere through improved OTU clustering. Environ Microbiol.

[75] Rognes T, Flouri T, Nichols B, Quince C, Mahé F (2016). VSEARCH: a versatile open source tool for metagenomics. PeerJ..

[76] McDonald D, Price MN, Goodrich J, Nawrocki EP, DeSantis TZ, Probst A (2012). An improved Greengenes taxonomy with explicit ranks for ecological and evolutionary analyses of bacteria and archaea. ISME J.

[77] Salter SJ, Cox MJ, Turek EM, Calus ST, Cookson WO, Moffatt MF (2014). Reagent and laboratory contamination can critically impact sequence-based microbiome analyses. BMC Biol.

[78] Schloss P, Westcott SL, Ryabin T, Hall JR, Hartmann M, Hollister EB (2009). Introducing mothur: open-source, platform-independent, community-supported software for describing and comparing microbial communities. Appl Environ Microbiol.

[79] Clarke KR (1993). Non-parametric multivariate analysis of changes in community structure. Aust J Ecol.

[80] Oksanen J, Blanchet FG, Friendly M, Kindt R, Legendre P, McGlinn D, Michin PR, O’Hara RB, Simpson GL, Solymos P, Stevens MHH, Szoecs E, Wagner H. vegan: Community Ecology Package. R package version 2.5–4. 2015.

[81] Team R development core. R. 2015.

