# Supplementary figures and images for: Similar bacterial communities on healthy and injured skin of black tip reef sharks
Source: Anim Microbiome. 2019 Sep 17;1:9. doi: 10.1186/s42523-019-0011-5 (PMC7807711; doi:10.1186/s42523-019-0011-5)

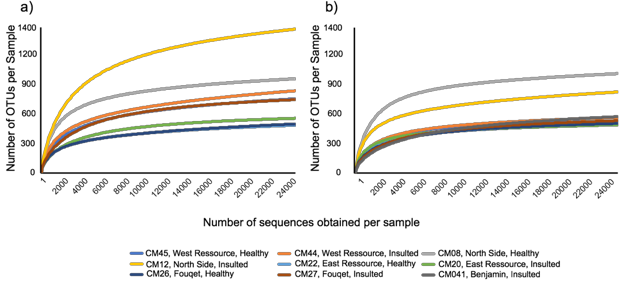

Supplement: Supplementary file 2 — Figure S1 Representative rarefaction curves for bacterial community sequencing efforts of healthy and insulted skin samples of black-tip reef sharks (Carcharhinus melanopterus) collected at five sites in the Amirante Islands, Seychelles. To facilitate presentation, one representative sample of a healthy and insulted skin sample is provided for each of the five sites, except for the site Benjamin, where no visibly healthy sharks could be sampled. a) Samples from skin around the gills, b) samples from skin on the back. Plateauing curves suggest adequate sequencing effort. (DOCX 704 kb) [file 42523_2019_11_MOESM2_ESM.docx]
