# Supplementary material for: Similar bacterial communities on healthy and injured skin of black tip reef sharks
Source: Anim Microbiome. 2019 Sep 17;1:9. doi: 10.1186/s42523-019-0011-5 (PMC7807711; doi:10.1186/s42523-019-0011-5)
Supplement: Supplementary file 6 — Table S3 Details on collection date, location, health state, and sex of individual blacktip reef sharks collected in 2017 around the Amirante Islands, Seychelles. Shark identifiers printed in bold indicate individuals with observed skin insult. (DOCX 19 kb) [file 42523_2019_11_MOESM6_ESM.docx]

| **No. of sharks** | **Date** | **No. of sharks / date** | **Island or Bank** | **Location** | **Sex** |
| --- | --- | --- | --- | --- | --- |
| 1 | 27/03/17 | 1 | St Joseph | West Ressource | female |
| 2 | 28/03/17 | 1 | St Joseph | West Ressource | female |
| **3** | **28/03/17** | **2** | **St Joseph** | **West Ressource** | **female** |
| 4 | 29/03/17 | 1 | St Joseph | West Ressource | female |
| **5** | **29/03/17** | **2** | **St Joseph** | **West Ressource** | **female** |
| **6** | **31/03/17** | **1** | **St Joseph** | **West Ressource** | **female** |
| **7** | **02/04/17** | **1** | **D'Arros** | **North Side** | **female** |
| 8 | 02/04/17 | 2 | D'Arros | North Side | male |
| 9 | 03/04/17 | 1 | D'Arros | North Side | female |
| 10 | 03/04/17 | 2 | D'Arros | North Side | female |
| 11 | 03/04/17 | 3 | D'Arros | North Side | female |
| **12** | **03/04/17** | **4** | **D'Arros** | **North Side** | **female** |
| 13 | 08/04/17 | 1 | D'Arros | North Side | female |
| 14 | 08/04/17 | 2 | D'Arros | North Side | female |
| 15 | 09/04/17 | 1 | D'Arros | North Side | female |
| 16 | 09/04/17 | 2 | D'Arros | North Side | male |
| 17 | 09/04/17 | 3 | D'Arros | North Side | female |
| 18 | 10/04/17 | 1 | St Joseph | West Ressource | male |
| 19 | 12/04/17 | 1 | St Joseph | East Ressource | male |
| **20** | **12/04/17** | **2** | **St Joseph** | **East Ressource** | **male** |
| **21** | **12/04/17** | **3** | **St Joseph** | **East Ressource** | **female** |
| 22 | 12/04/17 | 4 | St Joseph | East Ressource | female |
| 23 | 12/04/17 | 5 | St Joseph | East Ressource | male |
| 24 | 12/04/17 | 6 | St Joseph | East Ressource | female |
| 25 | 12/04/17 | 7 | St Joseph | East Ressource | male |
| 26 | 12/04/17 | 8 | St Joseph | Fouquet | male |
| **27** | **12/04/17** | **9** | **St Joseph** | **Fouquet** | **male** |
| 28 | 13/04/17 | 1 | St Joseph | East Ressource | female |
| 29 | 13/04/17 | 2 | St Joseph | East Ressource | male |
| 30 | 13/04/17 | 3 | St Joseph | East Ressource | female |
| **31** | **13/04/17** | **4** | **St Joseph** | **East Ressource** | **female** |
| 32 | 13/04/17 | 5 | St Joseph | Fouquet | male |
| 33 | 13/04/17 | 6 | St Joseph | Fouquet | female |
| 34 | 13/04/17 | 7 | St Joseph | Fouquet | male |
| 35 | 13/04/17 | 8 | St Joseph | Fouquet | female |
| **36** | **13/04/17** | **9** | **St Joseph** | **Fouquet** | **male** |
| **37** | **13/04/17** | **10** | **St Joseph** | **Benjamin** | **male** |
| **38** | **13/04/17** | **11** | **St Joseph** | **Benjamin** | **male** |
| **39** | **13/04/17** | **12** | **St Joseph** | **Benjamin** | **female** |
| **40** | **14/04/17** | **1** | **West Ressource** | **West Ressource** | **female** |
| **41** | **18/04/17** | **1** | **West Ressource** | **West Ressource** | **female** |
| 42 | 18/04/17 | 2 | West Ressource | West Ressource | female |
| 43 | 19/04/17 | 1 | West Ressource | West Ressource | female |
| **44** | **19/04/17** | **2** | **West Ressource** | **West Ressource** | **female** |
|  |  |  |  |  |  |
