# Supplementary material for: Similar bacterial communities on healthy and injured skin of black tip reef sharks
Source: Anim Microbiome. 2019 Sep 17;1:9. doi: 10.1186/s42523-019-0011-5 (PMC7807711; doi:10.1186/s42523-019-0011-5)
Supplement: Supplementary file 6 [file 42523_2019_11_MOESM3_ESM.docx]

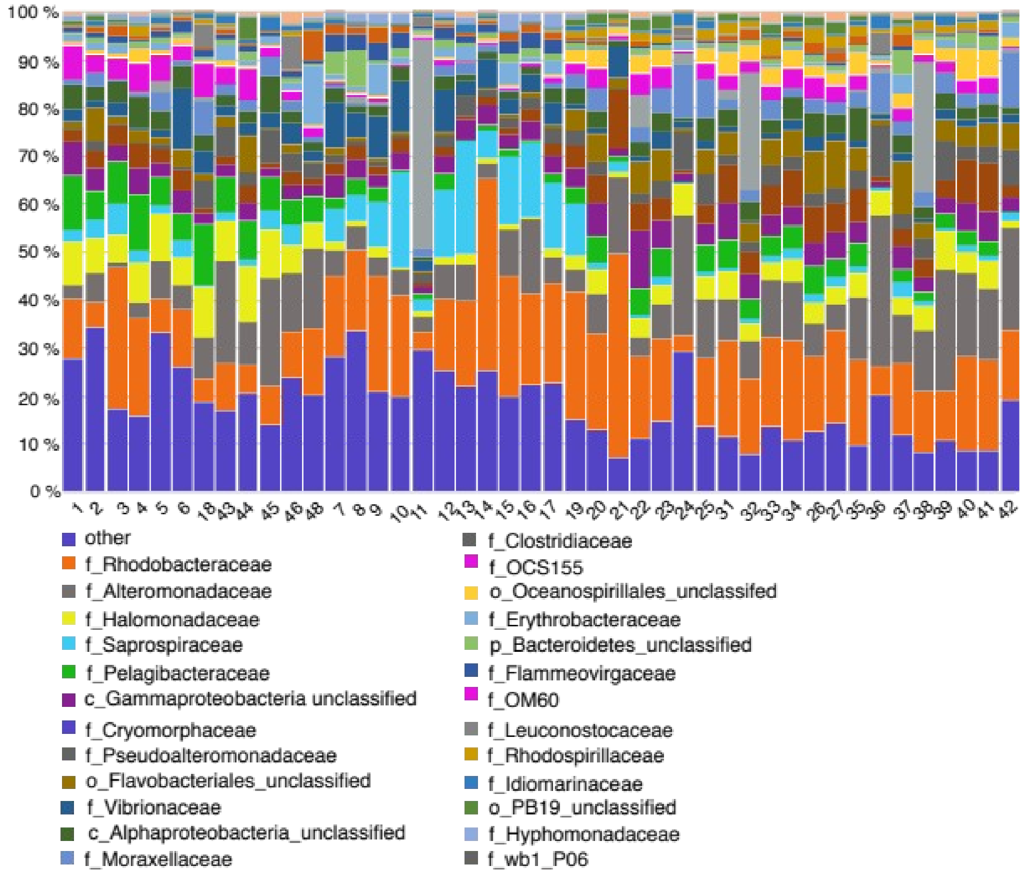
100 %
90 %
80 %
70 %
60 %
50 %
40 %
30 %
20 %
10 %
0 %
1 2 3 4 5 6 18 43 44 45 46 48 7 8 9 10 11 12 13 14 15 16 17 19 20 21 22 23 24 25 31 32 33 34 26 27 35 36 37 38 39 40 41 42
other
f_Rhodobacteraceae
f_Alteromonadaceae
f_Halomonadaceae
f_Saprospiraceae
f_Pelagibacteraceae
c_Gammaproteobacteria unclassified
f_Cryomorphaceae
f_Pseudoalteromonadaceae
o_Flavobacteriales_unclassified
f_Vibrionaceae
c_Alphaproteobacteria_unclassified
f_Moraxellaceae
f_Clostridiaceae
f_OCS155
o_Oceanospirillales_unclassifed
f_Erythrobacteraceae
p_Bacteroidetes_unclassified
f_Flammeovirgaceae
f_OM60
f_Leuconostocaceae
f_Rhodospirillaceae
f_Idiomarinaceae
o_PB19_unclassified
f_Hyphomonadaceae
f_wb1_P06

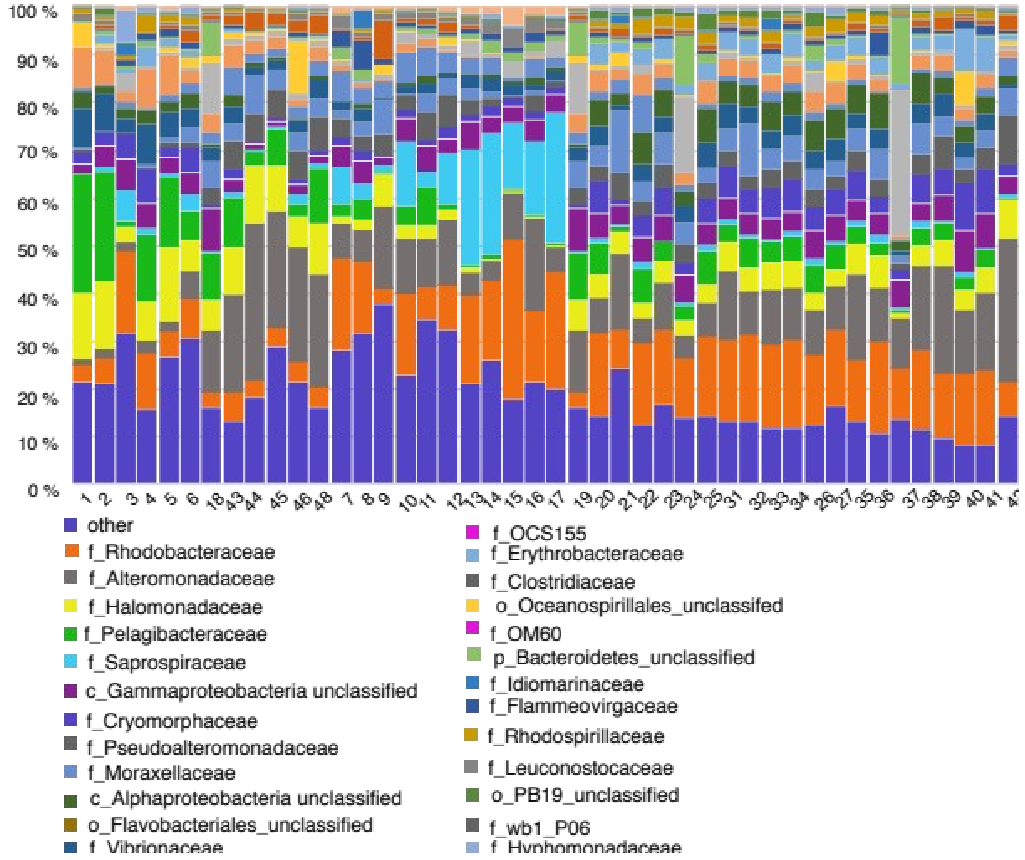
100 %
90 %
80 %
70 %
60 %
50 %
40 %
30 %
20 %
10 %
0 %
1 2 3 4 5 6 18 43 44 45 46 48 7 8 9 10 11 12 13 14 15 16 17 19 20 21 22 23 24 25 31 32 33 34 26 27 35 36 37 38 39 40 41 42
other
f_Rhodobacteraceae
f_Alteromonadaceae
f_Halomonadaceae
f_Pelagibacteraceae
f_Saprospiraceae
c_Gammaproteobacteria unclassified
f_Cryomorphaceae
f_Pseudoalteromonadaceae
f_Moraxellaceae
c_Alphaproteobacteria unclassified
o_Flavobacteriales_unclassified
f_Vibrionaceae
f_OCS155
f_Erythrobacteraceae
f_Clostridiaceae
o_Oceanospirillales_unclassifed
f_OM60
p_Bacteroidetes_unclassified
f_Idiomarinaceae
f_Flammeovirgaceae
f_Rhodospirillaceae
f_Leuconostocaceae
o_PB19_unclassified
f_wb1_P06
f_Hyphomonadaceae
